# Takagi–Sugeno Fuzzy Nonlinear Control System for Optical Interferometry

**DOI:** 10.3390/s25061853

**Published:** 2025-03-17

**Authors:** Murilo Franco Coradini, Luiz Henrique Vitti Felão, Stephany de Souza Lyra, Marcelo Carvalho Minhoto Teixeira, Claudio Kitano

**Affiliations:** Electrical Engineering Department, School of Engineering, São Paulo State University (UNESP), Ilha Solteira 15385-007, SP, Brazil; vit.felao@gmail.com (L.H.V.F.); stephany.lyra@unesp.br (S.d.S.L.); marcelo.minhoto@unesp.br (M.C.M.T.); claudio.kitano@unesp.br (C.K.)

**Keywords:** interferometry, fuzzy logic, Takagi–Sugeno, LMI, phase detection, nanodisplacement

## Abstract

The Takagi-Sugeno (T-S) fuzzy control is a nonlinear method that uses a combination of linear controllers as its control law. This method has been applied in various fields of scientific research: buck converters, biomedicine, civil engineering, etc. To the best of the authors’ knowledge, although works on traditional fuzzy control and optical interferometry have already been published, this is the first time that T-S fuzzy (specifically) is applied to demodulate interferometry signals. Through a proof-of-concept experiment, the paper describes the fusion of an open-loop interferometer with an external closed-loop digital observer based on T-S fuzzy (both simple and inexpensive), which actuates like a closed-loop interferometer (but without its drawbacks). The observer design is based on stability conditions using linear matrix inequalities (LMIs) solutions. The system is maintained at the optimal $90^{\circ }$ operation point (compensating for environmental drifts) and enables the demodulation of optical phase signals with low modulation index. Simulations and measurements were performed by using a Michelson interferometer, verifying that the method demodulates signals up to π/2 rad amplitudes and higher than 100 Hz frequencies (with maximum error of 0.45%). When compared to the important arc tangent method, both presented the same frequency response for the test PZT actuator.

## 1. Introduction

Control systems using Takagi–Sugeno (T-S) fuzzy logic [1] have a combination of linear controllers as the control law. The membership functions (denoted in this text as $\alpha_{ijl}$ and $\beta_{ijl}$) determine the extent to which each controller operates; these are obtained from the state variables at the instant the controllers are active.

The fundamentals of this control method, although well-established, still remain a subject of research since these allow innovations in several technological areas. Recently, studies have focused on relaxed stability conditions and applications of the method in increasingly different fields. However, in the specific area of this work—where the T-S fuzzy control is proposed for the demodulation of signals at the output of a nonlinear system, such as the interferometer—such problem-solving capacity has been scarcely explored.

Recent work has proven the success of the T-S method in studying the stability, design, and control technique of systems based on nonlinear buck converters [2]. In this line of application, T-S-fuzzy-based control was successfully applied to suppress the nonlinear effects of non-ideal components [3]. There is also a growing application of this method in studies related to biomedicine and human prosthetics. Based on a T-S fuzzy representation, an observer was designed by applying linear matrix inequality (LMI) techniques, using the frequencies at which the wheels are pushed to calculate the reference velocity of the center of gravity [4]. Simulation results confirm that the proposed control provides good maneuverability, enabling users to control the center of gravity’s velocity and the yaw rate of the wheelchair. Similar works can be found in [5,6]. In 2022, Nunes et al. proposed a robust control method, based on the T-S fuzzy model, applied to electrically stimulate the lower limbs [7]. The system was designed to handle uncertainties and non-idealities such as fatigue, spasms, tremors, and muscle recruitment.

In the study of structures (SHF—Structural Health Monitoring), a new approach was proposed to suppress the Sommerfeld effect (typical in the area of dynamic systems), which is a nonlinear phenomenon that occurs due to the interaction between non-ideal energy sources and a mechanical system operating in a non-stationary regime [8]. This suppression is achieved using T-S-fuzzy-based modeling, applied to a three-degree-of-freedom shear building model, and considering the equations of motion with and without nonlinear stiffness.

In the application of LMIs in optics, ref. [9] presents a robust control algorithm based on LMIs for the fiber optic voltage transformer (FOVT) to achieve optimized dynamic performance. The controller was designed to suppress uncertainties caused by temperature, thereby improving the dynamic performance of the fiber optic voltage transformer.

In [10], the same group introduces a closed-loop system for optical voltage sensors based on the Pockels effect, which is ideal for high-voltage applications. The LMIs yielded a controller capable of handling temperature variations of up to ±20%. Later, in [11], the group proposed an improvement for the fast dynamic tracking performance of the optical voltage sensor, the key difference being the use of the Lyapunov function as the theoretical foundation. In [12], a new closed-loop control system is proposed to optimize the detection accuracy and dynamic response characteristics of the integrated optical resonant gyroscope. The LMIs were used to develop a robust control algorithm that ensures system stability with $H_{\infty }$ performance, with the goal of improving the detection accuracy and dynamic performance of the gyroscope. More recently, in [13], a new system was developed to improve the temperature performance of the integrated optical resonant gyroscope, where LMIs were applied to determine the control gain used in the system.

Optical interferometry is a respectable technique recommended for measuring physical quantities whose sensors require extremely high sensitivity, such as detecting mechanical displacement amplitudes on the nano- or picometric scale [14]. Laser interferometry is noninvasive, has a wide bandwidth, and is compatible with fiber optic communication technology. Unlike traditional optical sensors, the interferometry information is imprinted in the phase (radians) of the light, and not in its optical intensity (W/m^2^).

A challenge in this area is that measurements are invariably influenced by spurious environmental disturbances. For example, temperature variations in the measurement area, caused by the heat of the sensor operator itself, or by vibrations generated by people walking or talking in the laboratory, can severely degrade the results (this phenomenon is called signal fading [15,16,17]), unless efficient signal processing or feedback control techniques are applied. It is important to emphasize that this drawback does not occur because interferometry is not efficient; rather, it occurs because it is extremely sensitive.

In addition, the transfer curve between the input (a given physical stimulus) and the output (a photo-detected electrical signal) of the interferometer has nonlinear behavior (sinusoidal dependence). This implies the existence of many both stable and unstable equilibrium points (under closed-loop operation), as well as the problem of phase ambiguity [18,19,20]. Thus, two major challenges of interferometry are related to performing measurements with great accuracy, in the face of unpredictable environmental variations and inherent nonlinear effects of the system.

To this day, when used, most closed-loop interferometry systems still employ the classic linear control, PID (proportional–integral–derivative) [14]. However, since this type of control generally requires piecewise linearization of the system (around certain stable equilibrium point), it is not very suitable for modern demands, where interferometers operate with a large dynamic range (deep-phase interferometry mode). In these cases, the use of passive optical phase-detection techniques such as PGC (phase-generated carrier) [21], Arctan [22], and others are strongly recommended, and sometimes closed-loop techniques are even discouraged [23].

Since the I/O relationship of the interferometer is nonlinear, it becomes natural to conjecture that a nonlinear control system is suitable for solving most problems in this area. Still, in the field of optics, in particular in interferometry, the number of applications of fuzzy logic is small, and that of T-S fuzzy is marginal. Even so, in 2007, a paper was published involving both fuzzy logic control (regular fuzzy, non T-S fuzzy) and optical interferometry [24]. In fact, a sensing path combining the advantages of an EFPI (extrinsic Fabry–Perot interferometer) and a PZT (piezoelectric material, with hysteresis) sensor was proposed to overcome the disadvantages when both (nonlinear) are used separately. As a global result, the intrinsic interferometric nonlinearity of a conventional EFPI sensor is compensated.

As a rule, the task of modeling measurement units applied to real-world problems and with a high level of precision is usually very laborious, due to the nonlinearities and dynamics inherent to every practical system [25].

One of the first works to propose a nonlinear control to compensate spurious influences on an optical nonlinear system was published by Zhu and Chen (1996), to compensate for the influence of the atmosphere and other drifts in a nonlinear double Fabry–Perot interferometer (including a piezoelectric actuator and its hysteresis effect) [26]. For this purpose, the PSHS (parallel subsystem of hardware simulation) technique was used; this is an effective engineering strategy for solving complex nonlinear systems whose responses constitute tasks that are too complicated to be deduced analytically.

In 2017, we proposed the use of nonlinear control theory, based on variable structure control and sliding modes (VS/SM), to interrogate interferometry sensors and obtained promising results, presenting important features such as ease of implementation, low cost, and robustness. To the best of our knowledge, we were pioneers in this control strategy. Thus, ref. [27] applied the technique to characterize the multi-axis piezoelectric flextensional actuator, using the bulk Michelson interferometer operating under low modulation depth. Sinusoidal signals and arbitrary waveforms were detected, and linearity, hysteresis, and frequency response (in module and phase) graphs were determined up to 20 kHz. However, a self-calibration process (see Section 2.1) of the interferometer was need, and its dynamic range was limited to π/2 rad. In [28], VS/SM was applied to a modified bulk Michelson interferometer to generate two-phase quadrature outputs, operating in the high-gain approach mode, and expanding the dynamic detection range of the method to values well above π/2 rad. The arrangement did not require reset procedures or initial self-calibration. Although the system presented excellent performance, it was necessary to employ an external optical phase modulator (PZT) in the feedback circuit to measure the value of the phase shift of interest (as inevitably occurs to every high-gain approach interferometry architecture). An important evolution of the previous method occurred in [29], in which the passive interferometry phase-detection method emulated the technique of the physical closed-loop high-gain approach. That is, the arrangement worked as it did in [28]; however, the interferometer operated in an open-loop form (significantly simplifying the optical hardware) and the external auxiliary modulator (PZT) was implemented digitally through the software (being able to assume very high gain values (rad/V)). Thanks to this, high-gain approach interferometry can reestablish its credibility, having previously been considered by many authors to be “not a practical approach” (mainly due to problems with the reset circuit). Regarding practical applications, the VS/SM technique was tested in the fiber optic version of the Michelson interferometer in the following research works: in [30], it was used to characterize a thin-film molybdenum-coated fiber optical phase modulator based on thermal effect; in [31], it was used to interrogate an opto-mechanical accelerometer; and in [32], it was used as a fiber optic seismometer based on a fiber optic Mach–Zehnder interferometer.

Given the impossibility of defining nonlinear systems that are accurate enough, a modern and successful solution refers to artificial intelligence (AI), including machine learning and deep learning [25]. Thus, the application of AI to a nonlinear system such as the interferometry seems to be a natural option. For example, ref. [33] used deep learning architectures to classify vibration patterns generated in a Michelson interferometer by handwriting on an optical desk, achieving an accuracy of 92% in identifying the characters a–z. Sokkar et al. (2022) used a Mach–Zehnder interferometer and artificial intelligence in the investigation of opto-thermo-mechanical features in the antimicrobial action of polyamide-6 fibers grafted by quaternary ammonium salt with nano zinc oxide [34]. The objective was to test non-traditional antimicrobial fabrics (a combination between antimicrobial agents and conventional polymeric fibers) for protection against microorganisms (*E. coli*, *S. aureus*, and *Candida albicans*) and prevent their spread. The mechanically drawn process (the draw ratio), the time, and the temperature of thermal annealing treatments were tested with the interferometer to improve the structural features of the fiber used in order to obtain the best antimicrobial activity. A convolutional neural network (a highly accurate, refined AlexNet–SVM network) was used to identify the predominant type of noise in the captured microinterferogram pattern and to specify the most appropriate type of band pass filter to perform the process of suppressing these kinds of noise. According to the network’s decision, the best way for denoising would be to apply a Wiener filter to all sampled microinterferograms. Once this was performed, the method chosen to detect the optical phase shift was the conventional arc tangent method. On the other hand, optical fiber interferometers combined with artificial intelligence techniques have been extensively used in intrusion detection systems, aiming to protect high-value assets and vital infrastructure such as refineries, petrochemical plants, government facilities, and military installations. In [35], a wide range of distributed fiber sensor networks—integrating a large number of sensors and with different purposes—are discussed, identifying phase-error-induced crosstalk and acquiring the ability to distinguish between normal building variations, false alarms, noise, and actual intrusions. Interferometry contributes with its high accuracy, responsiveness, and reliability. The number of applications of interferometry allied to AI and machine learning has been increasing significantly in recent years, as in the works of [36] (for classification of adulterant degree in liquid solutions), [37] (for free-form surface measurement), [38] (for optical metrology), and in several others, highlighting the impressive power and capacity of this tool.

In this scenario of detection methods using nonlinear control, below, considerations on the applications of the nonlinear fuzzy model to the nonlinear interferometry system are presented. Applications of fuzzy logic control to truly detect optical phase signals are rare, although it is possible to cite the work of Park and Song (2005), who used one of the first fuzzy versions for this purpose, the so-called fuzzy PID controller [39]. The proposal was used for a simple task (when compared with [24]), namely to stabilize the operating point of the interferometer against spurious environmental disturbances. Since the PID controller is not suitable for nonlinear systems, fuzzy logic was used, which has the ability to control nonlinear systems, eliminating some of the influences of environmental disturbances and allowing the interferometer to be stabilized and controlled. From then on, the detection technique used was simply the arc tangent. Fuzzy PID control was also used in 2015, for WLI (white light interferometry) for a similar application [40]. In 2020, an even simpler version, a fuzzy PI control, was used in a fiber optic current sensor (FOCS) [41]. Only the simulations have been published.

Conversely, the T-S Fuzzy strategy is significant because LMIs-based designs of controllers for T-S fuzzy models allows us to simultaneously consider uncertain parameters in plants, time delays, and input saturation. Doing so also enables us to specify performance indexes, such as decay rate (which is related to the setting time) and the $H_{\infty }$ or $H_{2}$ norms of the closed-loop system, such that the effect of a disturbance is attenuated. Furthermore, the design of the controllers is represented as a convex optimization problem that is not hard to solve [8,9]. It is worth mentioning that, in this paper, the design specification was only the asymptotic stability of the equilibrium point x = 0, using a simple quadratic Lyapunov function and the experimental results. In fact, there exist many other less conservative design methods that can be applied and improve the performance of this controlled system, such as those based on more sophisticated Lyapunov functions such as fuzzy Lyapunov functions [42,43], min type Lyapunov functions [44], and more elaborated control laws, such as switched controllers [45] (which may be tested in future publications). Despite this, the authors think that the procedure proposed here is a representative proof-of-concept example that opens new and important possibilities for designing powerful controllers for these systems.

As can be seen, the number of publications using T-S fuzzy nonlinear control (with or without LMIs) to solve the problems involved in interferometric phase detection are few, and, when found, they are not overarching or are produced by the same groups with incremental contributions to the same conception. In this work, the authors propose a novel method for detecting optical phase signals, immune to fading problems, and robust from the point of view of modern control theory. An important evolution is that the interferometer works in open loop mode, and all phase compensation is done by the external nonlinear observer system. The fusion of methods such as fuzzy T-S control theory and LMIs to measure microscopic displacements will be discussed. The theory, simulations, and experiments will be presented, and the efficiency of the technique will be tested by measuring displacements produced by a piezoelectric actuator positioned in one of the arms of a Michelson interferometer. Using very simple and inexpensive optical hardware, all the power of the method will be concentrated in the software. It will be shown that the proposed system is capable of competing with results as good as several proposals with complicated optics and electronics published in the literature.

## 2. Michelson Interferometer and T-S Fuzzy Theory

This section presents the foundation used for the development of this work and the implementation of the demodulation method. It will cover the theory of the classical and modified Michelson interferometer, the T-S fuzzy control method with stability analysis using Lyapunov, and, finally, the system model utilized.

### 2.1. Michelson Interferometer

**Figure 1:**
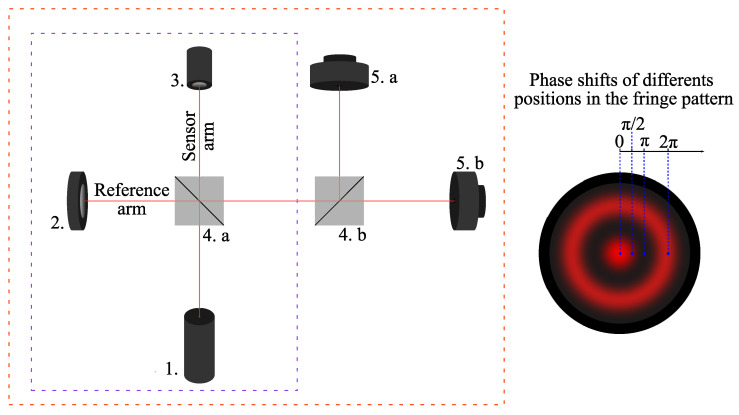
Modified Michelson interferometer.

In principle, a Michelson interferometer can be considered a standard instrument for displacement measurement due to its direct traceability to the primary standards of length [46]. The traditional Michelson interferometer corresponds to the drawing inside the dashed rectangle on the left side of Figure 1, composed of items 1, 2, 3, and 4.a [14]. Ideally, it operates as follows: The laser is the light source with a fixed wavelength, “λ”. The light beam reaches the beam splitter, where the laser beam is equally divided into two distinct beams. The reflected beam travels along the reference arm to a fixed mirror, which subsequently reflects it back toward the beam splitter. Similarly, the transmitted beam travels toward a mirror that undergoes positional fluctuations, thereby altering the length of the sensing arm. This beam then returns to the beam splitter, and both reflected beams (reference and sensing) proceed to the photodetector, where they produce a fringe pattern [29].

Following the strategy proposed by Felão et al. (2022) (who used VS/SM nonlinear control techniques), the implementation of a closed-loop nonlinear observer requires the generation of two quadrature phase terms (relative phase shift of π/2 rad) from a single interferometric output [29]. To obtain in-phase and quadrature signals, a simple modification can be made to the output of the traditional Michelson interferometer. After it reaches the beam splitter, the combined beam from the sensing and reference arms is subjected to another division at a second beam splitter (element 4.b), where both beams will exhibit a circular fringe pattern. These quadrature phase signals can be obtained by placing two photodetectors (elements 5.a and 5.b), each positioned in a specific region of the fringe pattern, as shown in Figure 1 [28].

After the suppression of the bias voltage [14], the output signals from the photodetectors, $PD_{1}$ and $PD_{2}$, are expressed by Equations (1) and (2):

(1) $$PD_{1}=A_{1}V_{1}\cos\left(\Delta \phi \left(t\right)+\phi_{0}\left(t\right)\right)\;\mathrm{and}$$

(2) $$PD_{2}=A_{2}V_{2}\sin\left(\Delta \phi \left(t\right)+\phi_{0}\left(t\right)\right),$$

where $A_{1}$ and $A_{2}$ are associated with the responsivity of the photodetectors and the gain of the transimpedance circuit. The terms $V_{1}$ and $V_{2}$ are referred to as fringe visibility, with values ranging between 0 and 1. Factors defining visibility include the parallelism between the electric field vectors of the combined beams, the beam intensities, and the coherence of the light source. The static phase shift, $\phi_{0}\left(t\right)$, is generated by the difference in the optical paths of the reference and sensing arms. However, this phase shift can fluctuate randomly over time (with frequencies below 20 Hz) due to environmental disturbances such as temperature variations, air turbulence, and mechanical vibrations. The term Δϕ(t) represents the signal of interest, related to the physical quantity being measured, by changing the optical path length of the sensing arm. In this work, it corresponds to the measurement of mechanical displacement generated by element 3 in Figure 1.

In the past, the term closed-loop interferometry was used to describe an interferometry sensor of physical magnitudes, in which a negative feedback circuit was used in order to achieve the following: (a) stabilize the quiescent operating point in the quadrature phase condition, and/or (b) convert the cosine I/O characteristic curve of the open-loop interferometer into a linear characteristic (in a high-gain approach—HGA) [14]. The problem with the HGA strategy was that the feedback loops, generally, were implemented externally to the interferometers, through optical, electronic, or optoelectronic hardware. Another drawback of the HGA was that the phase shift to be measured was not extracted directly at the interferometer output, but rather indirectly, from the voltage applied to an auxiliary optical phase modulator (usually a PZT or Pockels modulator), in order to implement the control law (mostly using a PID control). Because of this, a pre-calibration procedure of the phase modulator (in units of rad/V) was mandatory throughout the entire operating bandwidth.

The HGA has undergone an evolution after the work of Felão et al. (2022), since it now operates in an open-loop circuit and the physical phase modulator became a “virtual modulator” incorporated into the photo-detected signal processing system, thus generating an all-digital closed-loop observer [29]. By using the variable structure with sliding mode control system (VS/SM), the new sensor topology operates as passive interferometry (like the arc tangent or PGC methods); however, it benefits from all the advantages of the closed-loop system, being automatically controlled in real time, such as immunity to signal fading and feedback linearization of the cosine I/O characteristic curve.

Inspired by Felão et al. (2022) [29], in the present work, another all-digital closed-loop observer topology is being proposed: a passive interferometry phase-detection method that emulates a physical closed-loop detection system, but it uses the T-S fuzzy logic control technique instead of VS/SM control. The interferometer is operated in open-loop format (significantly simplifying the optical hardware) and the external auxiliary modulator (PZT) is implemented digitally (emulated) by software, inside the NI BNC 2090A (National Instruments, Austin, TX, USA) data acquisition board.

### 2.2. T-S Fuzzy Control Logic

Consider a nonlinear system described by

(3) $${\dot{x}}_{i}\left(t\right)=\sum_{j=1}^{n}f_{ij}\left(z\left(t\right)\right)x_{j}\left(t\right)+\sum_{k=1}^{m}g_{ik}\left(x\left(t\right)\right)u_{k}\left(t\right),i=1,\ldots ,n,$$

where $x_{j}\left(t\right)$, j=1,2,…,n represent the state variables, $u_{k}\left(t\right)$, k=1,2,…,m denote the inputs, and $z\left(t\right)={\left[z_{1}\left(t\right)\;z_{2}\left(t\right)\;\ldots \;z_{q}\left(t\right)\right]}^{T}$ is a vector whose the entries are the premise variables that depend on the state vector, x(t), and uncertain parameters or unknown variables. Supposing that the functions $f_{ij}(z\left(t\right)$, *i*, j=1,2,…,n, and $g_{ik}(z\left(t\right)$, k=1,2,…,m from (3) are bounded in a given operation region, there exists a systematic procedure for the exact representation of this nonlinear system by a T-S fuzzy model. This procedure, presented in [1,45,47], is based on the minimum and maximum values of the aforementioned system nonlinearities, $f_{ij}\left(z\left(t\right)\right)$ and $g_{ik}\left(z\left(t\right)\right)$; these are associated with a region of required operation in the state space and to the bounds of the uncertain parameters or unknown variables that compose the vector z(t). These bounds are defined in Equation (4), for all i,j=1,2,…,n and k=1,2,…,m:

(4) $$\begin{array}{c} a_{ij1}\equiv max\left(f_{ij}\left(z\left(t\right)\right)\right),\;\\ a_{ij2}\equiv min\left(f_{ij}\left(z\left(t\right)\right)\right),\;\\ b_{ik1}\equiv max\left(g_{ik}\left(z\left(t\right)\right)\right)\;\mathrm{and}\;\\ b_{ik2}\equiv min\left(g_{ik}\left(z\left(t\right)\right)\right). \end{array}$$

Now, consider the following definitions below, for all i,j=1,2,…,n and k=1,2,…,m:

(5) $$\begin{array}{c} \alpha_{ij1}\left(z\left(t\right)\right)=\frac{f_{ij}\left(z\left(t\right)\right)-a_{ij2}}{a_{ij1}-a_{ij2}},\;\\ \alpha_{ij2}\left(z\left(t\right)\right)=\frac{a_{ij1}-f_{ij}\left(z\left(t\right)\right)}{a_{ij1}-a_{ij2}},\;\\ \beta_{ij1}\left(z\left(t\right)\right)=\frac{g_{ik}\left(z\left(t\right)\right)-b_{ik2}}{b_{ik1}-b_{ik2}}\;\mathrm{and}\;\\ \beta_{ij2}\left(z\left(t\right)\right)=\frac{b_{ik1}-g_{ik}\left(z\left(t\right)\right)}{b_{ik1}-b_{ik2}}. \end{array}$$

where α and β are membership functions. The membership functions $\alpha_{1}\left(x_{1n}\left(t\right)\right)$ and $\alpha_{2}\left(x_{1n}\left(t\right)\right)$, presented in (5), define the extent to which each local model will act during control. During moments of change, such as disturbances or the establishment of the steady-state regime, the membership functions may vary rapidly. These oscillations occur because the system oscillates according to the membership function to reach the equilibrium point within the defined stability region, in this case, x = 0. Thus, the variation in the degrees of membership is directly linked to the system’s transient behavior, highlighting the importance of selecting appropriate membership functions [48]. In steady-state conditions, the variation between the degrees of membership enables a smooth transition between the two local models, resulting in a more stable and predictable system behavior.

Then, note that, from (4) and (5), it is possible to obtain exact representations of the nonlinear functions of the plant (3), in the given operation region, for all i,j=1,2,…,n and k=1,2,…,m: $f_{ij}\left(z\left(t\right)\right)=\alpha_{ij1}\left(z\left(t\right)\right)a_{ij1}+\alpha_{ij2}\left(z\left(t\right)\right)a_{ij2}$, $g_{ik}(z\left(t\right)=\beta_{ij1}\left(z\left(t\right)\right)b_{ik1}+\beta_{ij2}\left(z\left(t\right)\right)b_{ik2}$; accordingly, the important conditions $\alpha ij1\left(z\left(t\right)\right)+\alpha_{ij2}\left(z\left(t\right)\right)=1$, $\beta_{ij1}\left(z\left(t\right)\right)+\beta_{ij2}\left(z\left(t\right)\right)=1$ hold. Using these results, from the definitions in (5), an exact representation of the nonlinear system (3) by a T-S fuzzy model written in matrix form can be obtained, as shown in Equation (6) [1,45,47]:

(6) $$\dot{x}=\sum_{i=1}^{n}\sum_{j=1}^{n}\sum_{l=1}^{2}\alpha_{ijl}\left(z\left(t\right)\right)A_{ijl}x\left(t\right)+\sum_{i=1}^{n}\sum_{k=1}^{n}\sum_{l=1}^{2}+\beta_{ikl}\left(z\left(t\right)\right)B_{ikl}u\left(t\right),$$

where the entries ij of $A_{ijl}$ and $B_{ikl}$ are, respectively, equal to $a_{ijl}$ and $b_{ikl}$ for l=1,2, that were defined in (4), and all other entries of these two matrices are equal to 0. In the next sections, this procedure will be applied with details to obtain a T-S fuzy model and after to design a controller for an interferometer dynamical system. Finally, after some manipulations the system (6) can be described as the most-used T-S fuzzy model: $\dot{x}\left(t\right)=\left(\alpha_{1}\left(z\left(t\right)\right)A_{1}+\alpha_{2}\left(z\left(t\right)\right)A_{2}+\ldots +\alpha_{r}\left(z\left(t\right)\right)A_{r}\right)x\left(t\right)+\left(\alpha_{1}\left(z\left(t\right)\right)B_{1}+\alpha_{2}\left(z\left(t\right)\right)B_{2}+\ldots +\alpha_{r}\left(z\left(t\right)\right)B_{r}\right)u\left(t\right)$. Here, $\left(A_{i},B_{i}\right)$, i=1,2,…,n are known constant matrices called local models, and the conditions $\alpha_{1}\left(z\left(t\right)\right)$, $\alpha_{2}\left(z\left(t\right)\right)$, …,$\alpha_{r}\left(z\left(t\right)\right)$ ≥ 0, $\alpha_{1}\left(z\left(t\right)\right)+\alpha_{2}\left(z\left(t\right)\right)+\ldots +\alpha_{r}\left(z\left(t\right)\right)=1$ hold in the operation region.

#### 2.2.1. Stability Analysis via the Lyapunov Approach

In [1], theorems were established for stability analysis and control system design techniques for fuzzy systems, based on the direct Lyapunov method. Since the sampling frequency is considerably high, the discrete-time system closely resembles the continuous-time system, considering the last one for stability analysis. Equation (7) can be considered a candidate Lyapunov function, expressed as:

(7) $$V\left(x\left(t\right)\right)=x^{T}\left(t\right)Px\left(t\right)>0,\;\;x\left(t\right)\neq 0.$$

The stability study is summarized as finding a matrix P that is a positive definite, such that the time derivative of the Lyapunov function (V(x(t))) is negative, as shown in Equation (8):

(8) $$\dot{V}\left(x\left(t\right)\right)={\dot{x}}^{T}\left(t\right)Px\left(t\right)+x^{T}\left(t\right)P\dot{x}\left(t\right)<0,\;\;x\left(t\right)\neq 0.$$

Therefore, it is possible to establish a sufficient condition for the asymptotic stability of the equilibrium point x=0 of the open-loop system presented in Equation (3) (considering u(t)=0). Note that, considering the aforementioned exact T-S fuzzy model of (3), in this $\dot{x}\left(t\right)=\left(\alpha_{1}\left(z\left(t\right)\right)A_{1}+\alpha_{2}\left(z\left(t\right)\right)A_{2}+\ldots +\alpha_{r}\left(z\left(t\right)\right)A_{r}\right)x\left(t\right)$, where $\alpha_{1}\left(z\left(t\right)\right)$, $\alpha_{2}\left(z\left(t\right)\right)$, …,$\alpha_{r}\left(z\left(t\right)\right)\geq 0$ and $\alpha_{1}\left(z\left(t\right)\right)+\alpha_{2}\left(z\left(t\right)\right)+\ldots +\alpha_{r}\left(z\left(t\right)\right)=1$. Then, from (8) $\dot{V}\left(x\left(t\right)\right)={\dot{x}}^{T}\left(t\right)\left[\alpha_{1}\left(z\left(t\right)\right)\left(A_{1}^{T}P+{\mathrm{PA}}_{1}\right)+\alpha_{2}\left(z\left(t\right)\right)\left(A_{2}^{T}P+{\mathrm{PA}}_{2}\right)+\ldots +\alpha_{r}\left(z\left(t\right)\right)\left(A_{r}^{T}P+{\mathrm{PA}}_{r}\right)\right]x\left(t\right)$. Therefore, the conditions for the asymptotic stability of the equilibrium point, x=0, of this open-loop system can be described as the solution of the following problem, described by LMIs: find a symmetric positive definite matrix, P, common to the indexes, *i*, that satisfies the inequality (9) [1]:

(9) $$A_{i}^{T}P+PA_{i}<0,\;\;\;i=1,2,\ldots ,r.$$

Finding the matrix P has not always been a straightforward task, as early studies relied on trial-and-error procedures. In [49], this challenge was reduced to the problem of determining P for the stability analysis and a control design as a problem of linear matrix inequalities (LMIs), which can be efficiently solved using convex programming techniques.

The main idea behind using the concept of parallel-distributed compensation (PDC) is to design a controller for each rule of the T-S fuzzy model. For instance, in the case studied in this paper, the plant will be described as a T-S fuzzy model (as given in (16)) $\dot{x}\left(t\right)=\left(\alpha_{1}\left(z\left(t\right)\right)A_{1}+\alpha_{2}\left(z\left(t\right)\right)A_{2}\right)x\left(t\right)+\mathrm{Bu}\left(t\right)$, where B is a known constant matrix, $\alpha_{1}\left(z\left(t\right)\right)+\alpha_{2}\left(z\left(t\right)\right)=1$, $\alpha_{1}\left(z\left(t\right)\right)$, $\alpha_{2}\left(z\left(t\right)\right)\geq 0$ and the controller is given by $u\left(t\right)=-(\alpha_{1}\left(z\left(t\right)\right)F_{1}+\alpha_{2}\left(z\left(t\right)\right)F_{2})x$. From the perspective of each individual rule, the controllers are linear; however, the global system controller is nonlinear, as it results from the fuzzy combination of each individual controller.

Now, the PDC controller is defined, $u\left(t\right)=-(\alpha_{1}\left(z\left(t\right)\right)F_{1}+\alpha_{2}\left(z\left(t\right)\right)F_{2}+\ldots +\alpha_{r}\left(z\left(t\right)\right)F_{r})x$, where $F_{1}$, $F_{2}$, …,$F_{r}$ are constant gains.

A sufficient condition for the asymptotic stability of the equilibrium point x=0 of this controlled fuzzy system can be obtained using the Lyapunov function candidate (7), where $P=P^{T}>0$ and (8). The following conditions offer a solutions for this problem: find a symmetric positive definite matrix, P, common to all indexes, *l* and *k*, which satisfies the inequalities (10):

(10) $$\begin{array}{c} G_{ll}^{T}P+PG_{ll}<0\\ {\left(\frac{G_{lk}+G_{kl}}{2}\right)}^{T}P+P\left(\frac{G_{lk}+G_{kl}}{2}\right)\leq 0,\;l<k,\;k,l\;=1,2,\ldots ,r, \end{array}$$

where $G_{lk}=A_{l}-B_{l}F_{k}$ and $F_{k}$ comprise a gain matrix of the controller.

To determine the controller gains, $F_{k}$, we must substitute the value of $G_{lk}$ into (10) and multiply both sides by $X=P^{-1}=X^{T}$. This results in the LMI conditions shown in inequalities (11) and (12):

(11) $$XA_{l}^{T}-M_{l}^{T}B_{l}^{T}+A_{l}X-B_{l}M_{l}<0\;\mathrm{and}$$

(12) $$XA_{l}^{T}-M_{k}^{T}B_{l}^{T}+XA_{k}^{T}-M_{l}^{T}B_{k}^{T}+A_{l}X-B_{l}M_{k}+A_{k}X-B_{k}M_{l}\leq 0,\;l<k,$$

where $M_{l}=F_{l}X$ for all l∈{1,2,…,r}. Thus, using LMIs, the problem of designing the fuzzy controller to stabilize the system becomes finding the matrix X>0 and the matrices $M_{l}$, l=1,2,…,r that satisfy inequalities (11) and (12).

#### 2.2.2. T-S Fuzzy Modeling of the Interferometric Signal

**Figure 2:**
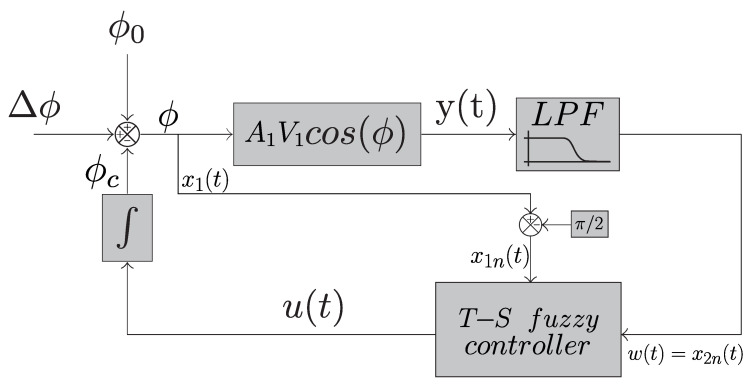
Block diagram of the interferometer dynamical system applied to the T-S fuzzy controller.

Considering the interferometer operating in open loop, whose photo-detected signal $PD_{1}$ (or $PD_{2}$) is described by (1) (or (2)), an observer system will be proposed, implemented in digital form, and driven by $PD_{1}$ (or $PD_{2}$). Figure 2 shows a sketch of the T-S fuzzy system working in closed loop within the observer. In this system, the low-pass filter (LPF) at the output of the block associated with the interferometer, $A_{1}V_{1}\cos\left(\phi \left(t\right)\right)$, where $\phi \left(t\right)=\Delta \phi \left(t\right)+\phi_{0}\left(t\right)+\phi_{c}\left(t\right)$, and whose output is y(t), implies that the fuzzy T-S control system must compensate only the low frequencies, related to the phase shift $\phi_{0}\left(t\right)$. In other words, this type of control follows the low-gain approach (LGA) [14], where the system will compensate only the low-frequency components generated by environmental drifts (20 Hz in this work), and will let the high-frequency components (Δϕ(t)) pass unchanged through the observer’s closed loop. Furthermore, if $\phi_{0}\left(t\right)$ is kept approximately constant around π/2 rad (region of greatest sensitivity and linearity of the interferometer), then the measurement (detection) of Δϕ(t) in the LGA condition becomes trivial. The chosen state variables will be $x_{1}\left(t\right)=\phi \left(t\right)=\Delta \phi \left(t\right)+\phi_{0}\left(t\right)+\phi_{c}\left(t\right)$ and $x_{2}\left(t\right)=w\left(t\right)$, where $\phi_{c}\left(t\right)$ is the correction phase, introduced by the control system in order to suppress variations in $\phi_{0}\left(t\right)$, and w(t) is the LPF output. The diagram in Figure 2 is for simulations; thus, the assumption that Δϕ(t) is initially known will be adopted (in the practical system, this value should be previously estimated). According to the fuzzy logic theory described in Section 2.2.1, in simplified terms, the fuzzy T-S control system applied to interferometry will work as follows: first, it will be necessary to define the state variables and the respective matrix system. Within the matrix system, an element containing a nonlinearity (represented below by $f_{21}$) will be found, which occurs due to the nonlinear nature of the interferometer itself. This function will depend on another state variable related to $x_{1}\left(t\right)$ (which will be called $x_{1n}$), and can be approximated by the sum of the maximum and minimum values weighted by membership functions. Thanks to this, the matrix system will also be represented by a sum between the matrices with the maximum and minimum values in its element that has the nonlinear function. Thus, the control law will be given by two linear controllers (related to the two linear models) also weighted by membership functions. To find this pair of controllers, LMIs will be used. The pair of linear controllers will be constant for the application of the fuzzy T-S system, while the membership functions will be variable and dependent on the state variable, $x_{1n}$. In this subsequent stage of the analysis, to define the control law, it will be necessary to have an estimate of the value of this variable.

As observed in Figure 2, the low-pass filter at the interferometer output (y(t)) indicates that the T-S fuzzy control system will compensate only for low frequencies ($\phi_{0}\left(t\right)$). Thus, a control approach based on the low-gain approach (LGA) is defined. This implies that the signal of interest will be accessed through the interferometric output rather than the control signal, as seen in high-gain approach (HGA) strategies. The presented T-S fuzzy system can be related to the one proposed in [27], which also employs a filter at the interferometer output and the low-gain approach. However, the control system proposed in that work utilized sliding mode theory instead of the T-S fuzzy control approach which is adopted here.

The low-pass filter used is described by the linear-time-invariant equation, $\dot{w}\left(t\right)=-\left(1/\tau \right)w\left(t\right)+\left(1/\tau \right)y\left(t\right)$, and has a cutoff frequency of 20 Hz, which typically corresponds to the maximum frequency of $\phi_{0}\left(t\right)$ (under laboratory environmental conditions, but it should be noted that the cutoff frequency can be adjusted for each environment).

The chosen state variables are $x_{1}\left(t\right)=\phi \left(t\right)=\Delta \phi \left(t\right)+\phi_{0}\left(t\right)+\phi_{c}\left(t\right)$ and $x_{2}\left(t\right)=w\left(t\right)$. With these state variables applied to the model in Figure 2 and introducing a variable change $x_{1n}\left(t\right)=x_{1}\left(t\right)-\pi /2$ and $x_{2n}\left(t\right)=x_{2}\left(t\right)$, one can obtain the following equations: ${\dot{x}}_{1n}\left(t\right)={\dot{x}}_{1}\left(t\right)=\dot{\phi }\left(t\right)=\Delta \dot{\phi }\left(t\right)+{\dot{\phi }}_{0}\left(t\right)+{\dot{\phi }}_{c}\left(t\right)$, and ${\dot{x}}_{2n}\left(t\right)=\dot{w}\left(t\right)=-\left(1/\tau \right)w\left(t\right)+\left(1/\tau \right)y\left(t\right)=-\left(1/\tau \right)w\left(t\right)+\left(1/\tau \right)A_{1}V_{1}\cos\left(\phi \left(t\right)\right)=-\left(1/\tau \right)x_{2}\left(t\right)-\left(1/\tau \right)A_{1}V_{1}\sin\left(x_{1n}\left(t\right)\right)$. Thus, defining the control input as $u\left(t\right)={\dot{\phi }}_{c}\left(t\right)$, the system can be represented in matrix form, as shown in Equation (13):

(13) $$\left[\begin{array}{c} {\dot{x}}_{1n}\left(t\right)\\ {\dot{x}}_{2n}\left(t\right) \end{array}\right]=\left[\begin{array}{cc} 0 & 0\\ -\frac{A_{1}V_{1}\sin\left(x_{1n}\left(t\right)\right)}{\tau x_{1n}\left(t\right)} & -\frac{1}{\tau } \end{array}\right]\left[\begin{array}{c} x_{1n}\left(t\right)\\ x_{2n}\left(t\right) \end{array}\right]+\left[\begin{array}{c} 1\\ 0 \end{array}\right]u\left(t\right)+\left[\begin{array}{c} 1\\ 0 \end{array}\right]\left(\Delta \dot{\phi }\left(t\right)+{\dot{\phi }}_{0}\left(t\right)\right).$$

The nonlinear function was obtained in the modeling stage of the interferometry system, being present in the term $a_{21}$ in the state matrix **A** from Section 2.2, which is equivalent to $f_{21}\left(x_{1n}\left(t\right)\right)=-\frac{A_{1}V_{1}\sin\left(x_{1n}\left(t\right)\right)}{\tau x_{1n}\left(t\right)}$. Next, the nonlinear system (13) will be exactly represented by a T-S fuzzy model. A method of representing this matrix is to use local linear models, as described at the beginning of Section 2.2. The number of models required for this representation is $2^{k}$, where *k* is the number of nonlinear elements in the dynamic system. In the studied case, k=1. The local models are obtained based on the minimum and maximum values of the nonlinear function within a range of interest (in this work, −20π<ϕ<20π).

Based on the method presented at the beginning of Section 2.2, using Equations (4)–(6), the nonlinear function $f_{21}\left(x_{1n}\left(t\right)\right)$ can be expressed as the sum of the minimum and maximum values weighted by the membership functions, $\alpha_{1}\left(x_{1n}\left(t\right)\right)$ and $\alpha_{2}\left(x_{1n}\left(t\right)\right)$, respectively, as shown in Equation (14):

(14) $$f_{21}\left(x_{1n}\left(t\right)\right)=\alpha_{1}\left(x_{1n}\left(t\right)\right)\bar{f}+\alpha_{2}\left(x_{1n}\left(t\right)\right)\underline{f},$$

where $\underline{f}=min\left(f_{21}\left(x_{1n}\left(t\right)\right)\right)$ and $\bar{f}=max\left(f_{21}\left(x_{1n}\left(t\right)\right)\right)$. Note that $\alpha_{1}\left(x_{1n}\left(t\right)\right)$, $\alpha_{2}\left(x_{1n}\left(t\right)\right)\geq 0$ and $\alpha_{1}\left(x_{1n}\left(t\right)\right)+\alpha_{2}\left(x_{1n}\left(t\right)\right)=1$.

By performing some manipulations, the factors $\alpha_{1}$ and $\alpha_{2}$ can be rewritten as shown in Equation (15):

(15) $$\begin{array}{cc} \alpha_{1}\left(x_{1n}\left(t\right)\right)=\frac{\bar{f}-f_{21}\left(x_{1n}\left(t\right)\right)}{\bar{f}-\underline{f}} & \alpha_{2}\left(x_{1n}\left(t\right)\right)=\frac{f_{21}\left(x_{1n}\left(t\right)\right)-\underline{f}}{\bar{f}-\underline{f}}. \end{array}$$

Thus, the nonlinear system can be expressed as shown in Equation (16), with the state matrices defined in Equations (17):

(16) $$\begin{array}{c} \dot{x}\left(t\right)=\left(\alpha_{1}\left(x_{1n}\left(t\right)\right)A_{1}+\alpha_{2}\left(x_{1n}\left(t\right)\right)A_{2}\right)x\left(t\right)+\left(\alpha_{1}\left(x_{1n}\left(t\right)\right)B_{1}+\alpha_{2}\left(x_{1n}\left(t\right)\right)B_{2}\right)u\left(t\right)+\ldots \\ \ldots +\left(\alpha_{1}\left(x_{1n}\left(t\right)\right)D_{1}+\alpha_{2}\left(x_{1n}\left(t\right)\right)D_{2}\right)\left(\Delta \dot{\phi }\left(t\right)+{\dot{\phi }}_{0}\left(t\right)\right), \end{array}$$

(17) $$\begin{array}{cccc} A_{1}=\left[\begin{array}{cc} 0 & 0\\ \underline{f} & -\frac{1}{\tau } \end{array}\right] & A_{2}=\left[\begin{array}{cc} 0 & 0\\ \bar{f} & -\frac{1}{\tau } \end{array}\right] & B_{1}=B_{2}=B=\left[\begin{array}{c} 1\\ 0 \end{array}\right] & D_{1}=D_{2}=D=\left[\begin{array}{c} 1\\ 0 \end{array}\right] \end{array}.$$

**Figure 3:**
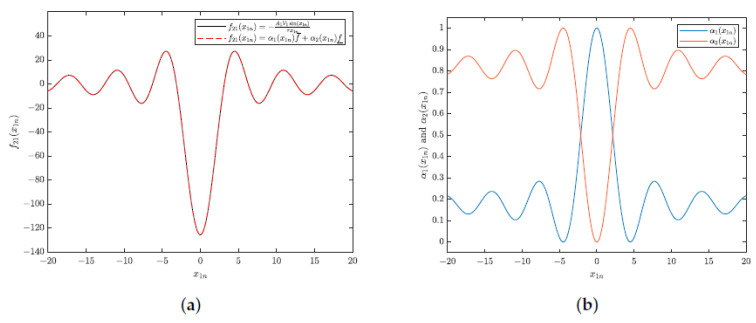
Graphics of nonlinear function of $x_{1n}\left(t\right)$ (Equation (14)) and membership functions (Equation (15)) in region of interest (−20 ≤ $x_{1n}\left(t\right)$ ≤ 20). (**a**) Nonlinear function of $x_{1n}\left(t\right)$. (**b**) Membership functions.

By varying the value of $x_{1n}\left(t\right)$ from −20 to 20 radians, and considering $A_{1}V_{1}=1$ and τ=1/40π, the function $f(x_{1n}\left(t\right))$ and the functions of membership ($\alpha_{1}\left(x_{1n}\left(t\right)\right)$ and $\alpha_{2}(x_{1n}\left(t\right))$) are illustrated in Figure 3.

In the controlled system, the input of the T-S fuzzy model (16) and (17) is given by $u\left(t\right)=-(\alpha_{1}\left(x_{1n}\left(t\right)\right)F_{1}+\alpha_{2}\left(x_{1n}\left(t\right)\right)F_{2})x\left(t\right)$, where $\alpha_{1}\left(x_{1n}\left(t\right)\right)$ and $\alpha_{2}\left(x_{1n}\left(t\right)\right)$ are described in (15) and $F_{1}$, $F_{2}$ are constant gains. It was developed and designed on the basis of the stability conditions of the T-S fuzzy systems, employing solutions through LMIs. Using MATLAB (R2022b) with the YALMIP toolbox (R20180612) and the LMILAB solver, a script was created to design the fuzzy control system. Using inequalities (11) and (12), the system of inequalities (LMIs) was constructed. The script confirmed the feasibility of the system, thus proving its stability for the gains $F_{1}=\left[-41.816108072133450,-2.743721401032494\times 10^{4}\right]$ and $F_{2}=\left[9.625586760137379,6.015949786742760\times 10^{3}\right]$. It is worth noting that MATLAB/YALMIP is really useful in designing LMI-based controllers; we only need the MATLAB/YALMIP solver to obtain the gains $F_{1}$ and $F_{2}$. Once that is settled, the control system can be applied to various platforms, including real-time systems, FPGA platforms, embedded systems, or even custom hardware.

## 3. Simulation of the T-S Fuzzy Control Applied to the Michelson Interferometer

**Figure 4:**
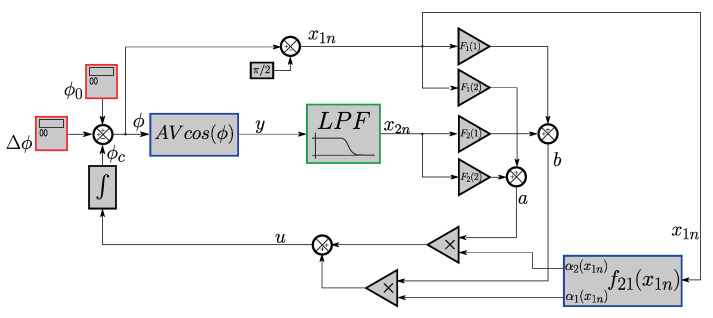
Interferometer control system using fuzzy logic employed in simulation.

In this section, the computational simulation of the system described so far will be discussed. First, the system was simulated using SIMULINK software (R2022b) in a closed-loop configuration, following the diagram in Figure 4, which consists of the following: two signal generators (red blocks) to generate Δϕ(t) and $\phi_{0}\left(t\right)$; two blocks of functions (blue blocks), one containing the interferometer function and another block determining $\alpha_{1}$ and $\alpha_{2}$ based on the value of $x_{1n}\left(t\right)$; the low-pass filter shown in Figure 2 (green block); the gains with the previously determined values $F_{1}=\left(F_{1}\left(1\right),F_{1}\left(2\right)\right)$ and $F_{2}=\left(F_{2}\left(1\right),F_{2}\left(2\right)\right)$; and finally, the integrator in the system’s feedback loop. The simulation was conducted in discrete time, where the discrete-time transfer function of the filter was obtained using the bilinear transformation.

The terms *A* and *V* will be considered as 1, once the interferometric output signal is normalized (as seen later in this section).

The simulations performed using SIMULINK had a sampling frequency of $f_{s}=10^{6}\;$Hz, with a time interval of 0.15 s The spectral analyses presented in the simulations use a frequency resolution of 0.5 Hz, applying a Hanning window for signal tapering. The same windowing method was adopted for the spectral analysis of the experimental results.

**Figure 5:**
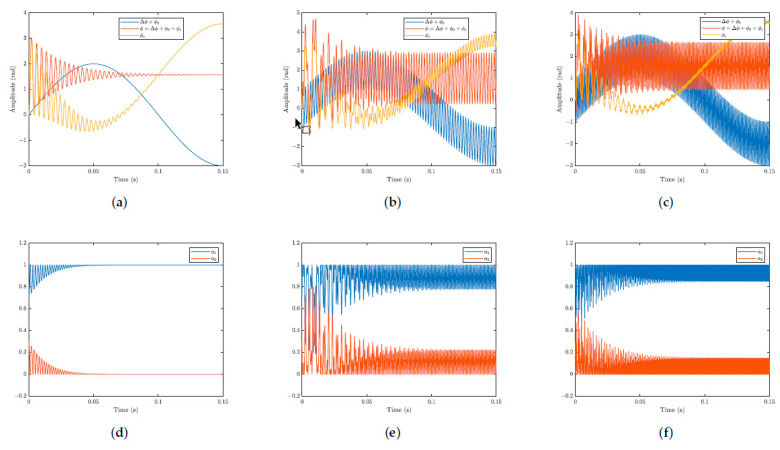
Simulation results, system temporal response, and variation of membership functions over time. (**a**) Temporal response for (Δϕ(t)=0) and $\phi_{0}\left(t\right)=2\sin\left(2\pi \times 5\times t\right)$. (**b**) Temporal response for Δϕ(t)=sin(2π×500×t)) and $\phi_{0}\left(t\right)=2\sin\left(2\pi \times 5\times t\right)$. (**c**) Temporal response for Δϕ(t)=sin(2π×1000×t) and $\phi_{0}\left(t\right)=2\sin\left(2\pi \times 5\times t\right)$. (**d**) functions of membership for (Δϕ(t)=0) and $\phi_{0}\left(t\right)=2\sin\left(2\pi \times 5\times t\right)$. (**e**) Functions of membership for Δϕ(t)=sin(2π×500×t)) and $\phi_{0}\left(t\right)=2\sin\left(2\pi \times 5\times t\right)$. (**f**) Functions of membership for Δϕ(t)=sin(2π×1000×t) and $\phi_{0}\left(t\right)=2\sin\left(2\pi \times 5\times t\right)$.

In Figure 5, the graphs in the first row show the input ($\Delta \phi \left(t\right)+\phi_{0}\left(t\right)$), the temporal response of the system (ϕ(t)), and the phase correction ($\phi_{c}\left(t\right)$), while the graphs in the second row show the variation of membership functions over time ($\alpha_{1}$ and $\alpha_{2}$). Figure 5a,d illustrate the simulations where the signal of interest (Δϕ(t)) was not included, with only the low-frequency signal ($\phi_{0}\left(t\right)=2\sin\left(2\pi \times 5\times t\right)$) as input. In Figure 5b,e, the simulations are illustrated with the input as a combination of high-frequency (Δϕ(t)=sin(2π×500×t)) and low-frequency ($\phi_{0}\left(t\right)=2\sin\left(2\pi \times 5\times t\right)$) signals. In Figure 5c,f, the system input was the combination of the signal Δϕ(t) (Δϕ(t)=sin(2π×1000×t)) with the same signal $\phi_{0}\left(t\right)$ ($\phi_{0}\left(t\right)=2\sin\left(2\pi \times 5\times t\right)$) already used in the other simulations. In both simulations the amplitude of the environmental disturbance term $\phi_{0}$ was considered severe, equal to twice the amplitude of the signal of interest.

In the first column of graphs in Figure 5a,d, it can be observed that, in a simulation without a high-frequency signal, the system converges to the equilibrium point $x_{1}\left(t\right)=\pi /2$. However, it is important to note that a variable transformation was performed, where the point $x_{1n}\left(t\right)$ converges to 0 ($x_{1n}\left(t\right)=x_{1}\left(t\right)-\pi /2$). Since there is no signal with a frequency higher than the filter’s cutoff frequency, the system remains fixed at the equilibrium point. The membership functions oscillate until they reach the equilibrium point and then remain constant.

The second and third columns of graphs in Figure 5b,c,e,f show that, when the input includes the high-frequency signal in addition to the low-frequency signal, the output signal exhibits only the high-frequency component oscillating around the equilibrium point $x_{1n}\left(t\right)$. The correction signal $\phi_{c}\left(t\right)$ contains the low-frequency component. After the transient phase, the degree of membership oscillates at the frequency Δϕ(t) between the two controllers.

**Figure 6:**
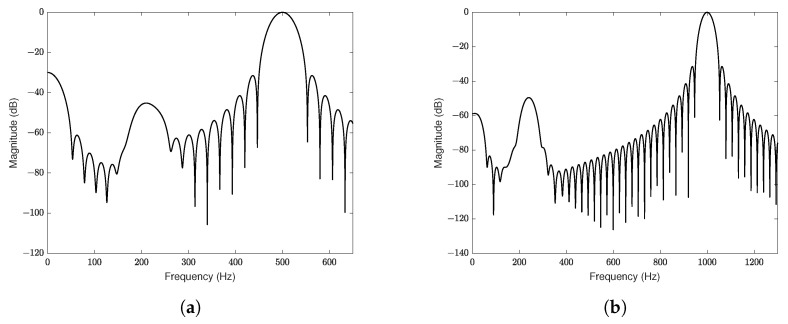
Spectral analysis of the system output. (**a**) Input at 500 Hz. (**b**) Input at 1000 Hz.

To demonstrate the frequency of the output signals in Figure 5b,c, a spectral analysis of the signal was performed between 0.075 and 0.15 s and is shown in Figure 6. The spectral analysis for Δϕ(t) with a frequency of 500 Hz is shown in the first graph (Figure 6a), and for a frequency of 1000 Hz in the second graph (Figure 6b).

Since the amplitude of $\phi_{0}\left(t\right)$ was chosen to be twice the amplitude of Δϕ(t) in Figure 5, from the spectral analyzes shown in Figure 6, it is evident that the signal $\phi_{0}\left(t\right)$ is compensated, allowing the predominance of the signal Δϕ(t) in the demodulated output. Actually, the side lobes—present below the signal frequency, Δϕ(t)—are associated with the transient regimes of the signals shown in Figure 5.

**Figure 7:**
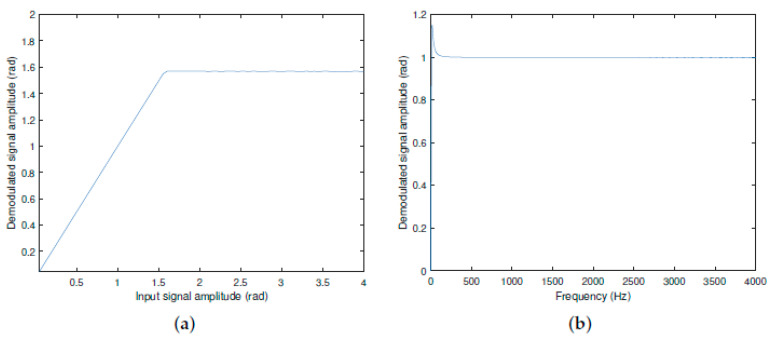
Dynamic range sweep of the T-S fuzzy method. (**a**) Amplitude sweep. (**b**) Frequency sweep.

Amplitude and frequency sweeps were also performed using the LABVIEW software (version 15.0). The system used is the same as the one used experimentally, as will be shown in the next section (Figure 9b). The system’s input consisted of sine waves generated by the software, with amplitudes and frequencies defined in the program. In Figure 7a, the result of the amplitude sweep is shown, where the input signal frequency was kept constant at 500 Hz, and the amplitude was varied from 0.05 to 4 rad in steps of 0.05 rad. In Figure 7b, a frequency sweep was performed by varying the input signal frequency while keeping the amplitude fixed at 1 rad. The frequency range varied from 10 to 4000 Hz in steps of 1 Hz.

In Figure 7a, it can be observed that the method operates up to π/2 rad peak (1.57 rad peak), which is the limit of the arc cosine function’s range (see Section 4). Beyond this value, the phase-detected signal becomes wrapped, but the information should be recovered by unwrapping techniques. Consequently, the dynamic range of this T-S fuzzy method ranges from 0 to π/2 rad peak (not taken into account the noise). In Figure 7b, the frequency sweep indicates that there is a peak at 15 Hz, the value of which is 1.15211 radians; then, the curve enters a flat region, which is recommended for reliable measurements. Thus, in principle, the T-S fuzzy method would become effective above 15 Hz (according to next paragraphs, the choice this lower limit must be increased due to practical reasons).

**Figure 8:**
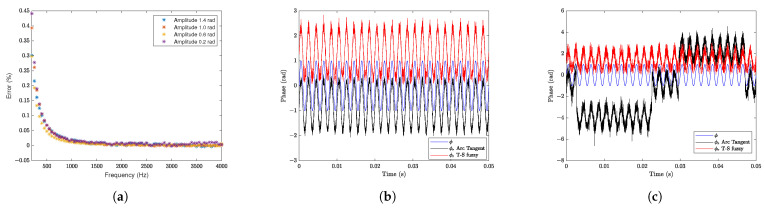
Error and noise sensitivity analysis of the fuzzy T-S method. (**a**) Relative error obtained by the fuzzy T-S method from 100 Hz to 4000 Hz. (**b**) Sensitivity to noise with a standard deviation of 0.1 (SNR = 15 dB). (**c**) Sensitivity to noise with a standard deviation of 0.3 (SNR = 5 dB).

In the following, a more complete analysis of the error and noise sensitivity was carried out, and the results were obtained via simulation. In Figure 8a, we illustrate the percentage error in the measurement of sinusoidal signals with amplitudes of 0.2, 0.6, 1.0, and 1.4 radians, at frequencies from 100 Hz to 4000 Hz. The largest error in this frequency band occurs near the lower limit and is 0.45%. This band was selected precisely because it is a favorable region where the error is less than 0.45% at 100 Hz. Despite the accelerated growth of the curve at the lower limit of the band, the maximum error at 15 Hz is only 13%. This behavior occurs due to the slight amplification of the system observed in the spectrum of Figure 7b. In turn, by considering white noise in the interferometry outputs, the response of the T-S fuzzy system was analyzed as shown in Figure 8b and Figure 8c, respectively. Sinusoidal input signals with 1.0 rad amplitude at 500 Hz were considered (in blue color), for additive noises with standard deviations of 0.1 (SNR = 15 dB) and 0.3 (SNR = 5 dB); the noisy output signals, obtained by the arc tangent and T-S fuzzy methods, are shown in black and red colors, respectively. Regarding noise sensitivity, the fuzzy T-S method demonstrated higher immunity. This is because the arc tangent method is subject to phase ambiguity, and may experience phase jumps (of 2π rad intervals) with a simultaneous abrupt change in the signal (and consequently, periodic errors). Actually, even if the input signal is smaller than π/2 rad, when it is in the presence of additive noise, it can generate spikes that exceed π/2; therefore, it can produce a totally incorrect phase detection. This occurs because the phase unwrapping routine of the arc tangent method inadvertently generates a phase jump of 2π radians. This type of mistake is significantly evident in Figure 8c. This drawback does not occur in the fuzzy T-S method; since the system has only one stable equilibrium point, the observer’s closed loop always keeps the signal at the same operating point.

Since these results do not take into account only the action of the low-pass filter with a cutoff frequency of 20 Hz (frequency below which the fuzzy T-S method should compensate for random environmental variations), we prefer to choose the beginning of the dynamic range at 100 Hz, corresponding to the maximum error of 0.45%, in accordance with Figure 8a.

As revealed in the text, the nonlinear fuzzy T-S method proposed in this work provides a single stable equilibrium point, around the phase quadrature condition, $\phi_{0}=\pi /2$ rad. This may constitute a disadvantage in relation to other nonlinear control methods, such as VS/SM in LGA mode (with presents many stable equilibrium points), for example [27]. In fact, if the sensor is subjected to an intense external disturbance, capable of moving it far away from the stable equilibrium point, the observer may have some difficulty in bringing it back to normal operation quickly enough, despite very high controller gains can be provided when the phase compensation modulator is implemented in software (instead with PZTs). On the other hand, this architecture is still advantageous compared to VS/SM, since it does not present problems such as inconvenient chattering, and so, it obtains better accuracy [27,28,29].

## 4. Experimental Setup

**Figure 9:**
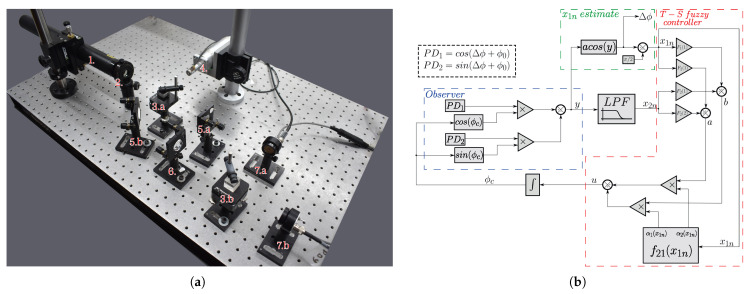
Physical and digital system of the experiment. (**a**) Experimental setup. (**b**) Block diagram of the T-S fuzzy control.

This section discusses the experimental setup, shown in Figure 9a, where a modified open-loop Michelson interferometer (Figure 1) is used. As observed in simulation, in principle, the method does not require two quadrature signals; however, since it was developed within the observer framework described in [29], the quadrature formed by the modified Michelson interferometer is necessary. (To apply the method without signal quadrature, a second piezoelectric actuator would be required in place of the fixed mirror in the reference arm to enable physical feedback within the system [28]). The experimental setup shown in Figure 9a consists of the following components: a He-Ne laser operating at λ=632.8nm with a power of 5 mW (1); a polarizer to prevent feedback into the laser cavity (2); two beam splitters (CMI-BS013 from Thorlabs, Newton, NJ, USA)—one to split the beams between the sensing and reference arms (3.a), and the other to divide the beams for the photodetectors forming the quadrature signals (3.b); a commercial piezoelectric actuator (PZT, Control Technics, Powys, UK), responsible for generating the Δϕ(t) signal; two mirrors—one to reflect the beam in the reference arm (5.a) and the other to change the beam’s direction (5.b); a negative lens to expand the beam area, facilitating the quadrature condition adjustment (6); two photodetectors (PD_1_ and PD_2_) to interrogate the interferometric signals (7.a and 7.b). The photodetectors are PIN photodiodes of the PDA 55 model (Thorlabs, Newton, NJ, USA).

In Figure 9b, the block diagram implemented in LABVIEW is shown. Here, $PD_{1}$ and $PD_{2}$ represent the interferometric signals after normalization, which removes the AV terms noted in Equations (1) and (2), leaving only the cosine and sine components. The schematic shows the blocks of the T-S fuzzy system working in a closed loop inside the observer, which works as a “digital” closed loop inside the LABVIEW environment. Since the T-S fuzzy system needs the interferometric output, $y\left(t\right)=\cos(\Delta \phi \left(t\right)+\phi_{0}\left(t\right)+\phi_{c}\left(t\right))$, it must be synthesized by the various blocks shown in the diagram. The block described in Figure 4 to determine the membership functions is also shown. As previously stated, an estimate of the value of $x_{1n}$ is necessary: such an initial estimate can be obtained from $x_{1}$, where the signal of interest is obtained by the arc cosine function shown in the diagram. From there, an estimate for $x_{1n}$ is also obtained; with this estimated value, it is possible to determine the values of the membership functions at that instant. By applying the control law, it is possible to close the loop inside the observer, compensating $\phi_{0}\left(t\right)$ and obtaining the signal of interest Δϕ(t) through the port of the arc cosine function.

Since the system operates within the observer framework, the output of the “virtual interferometer” is expressed as the sum of the products $y\left(t\right)=\cos\left(\Delta \phi \left(t\right)+\phi_{0}\left(t\right)+\phi_{c}\left(t\right)\right)=\cos\left(\Delta \phi \left(t\right)+\phi_{0}\left(t\right)\right)\cos\left(\phi_{c}\left(t\right)\right)-sin\left(\Delta \phi \left(t\right)+\phi_{0}\left(t\right)\right)sin\left(\phi_{c}\left(t\right)\right)$ (blue dashed rectangle in Figure 9b). The output is processed using the arc cosine function to obtain $x_{1}\left(t\right)$. A subsequent variable transformation is performed to compute $x_{1n}\left(t\right)$, defined as $x_{1n}\left(t\right)$ ($x_{1n}\left(t\right)=x_{1}\left(t\right)-\pi /2$) (green dashed rectangle in Figure 9b). Actually, it is possible to use the arc cos function to estimate $x_{1n}$, since the system operates at a low modulation index (amplitudes up to π/2). With the operating point at π/2, there would be no issue with the limitation of the image of the arc cos function (Im(arccos)=[0,π]). Using the value of $x_{1n}\left(t\right)$, a mathematical function block calculates the membership degree values ($\alpha_{1}$ and $\alpha_{2}$) at that moment in time, *t*. The same output y(t) is passed through a low-pass filter, and the filter output defines $x_{2n}\left(t\right)$. The values of gains $F_{1}$ and $F_{2}$—previously determined in Section 2.2.2—are then applied to $x_{1n}\left(t\right)$ and $x_{2n}\left(t\right)$; their corresponding terms are summed as follows: ($a=x_{1n}\left(t\right)F_{1}\left(1\right)+x_{1n}\left(t\right)F_{2}\left(1\right)$ and $b=x_{1n}\left(t\right)F_{1}\left(2\right)+x_{1n}\left(t\right)F_{2}\left(2\right)$). With the functions of membership values $\alpha_{1}$ and $\alpha_{2}$ determined as previously described, the input variable u(t) is calculated as $u\left(t\right)=\alpha_{1}a+\alpha_{2}b$. The functions of membership determine the extent to which the gains $F_{1}$ and $F_{2}$ influence the system (red dashed shape in Figure 9b).

It is important to highlight that, besides producing the variable $x_{1}$, the diagram in Figure 9b reveals that the arc cos(y) block has two other purposes: (a) it is in this access that the final output signal, Δϕ(t), of the compensated system is measured (it is in this port that the interferometric signal is extracted); (b) concomitantly, this is also the place where the estimated value of Δϕ(t) is inserted into the observer system.

To further analyze Figure 9b, in an ideal scenario where the environmental disturbance $\phi_{0}\left(t\right)=0$, during the first cycle of data acquisition, the initial conditions are considered to be zero. In this case, the estimated variable is perfect, being equal to the information ($x_{1n}=\Delta \phi \left(t\right)$), making it evident that the system reaches π/2. The correction phase ($\phi_{c}\left(t\right)$) is equal to zero, since $\phi_{0}\left(t\right)=0$. However, in practical conditions, the environmental disturbance is non-zero ($\phi_{0}\left(t\right)\neq 0$). Consequently, the estimated variable has an error ($x_{1n}\neq 0$). In parallel with the sliding mode system designed in [27], the $F_{1}$ and $F_{2}$ gains are sufficiently large (as determined by the feasibility analysis conducted earlier) to compensate for $\phi_{0}\left(t\right)$. The membership functions transition smoothly from 0 to 1. Unlike the sign function, which causes an abrupt transition in control with sliding modes, the membership functions in the T-S fuzzy control system continuously adjust to determine the optimal controller. In other words, they adapt the gains dynamically, ensuring that the estimated error is corrected and the system is stabilized without introducing oscillations in the control signal (such as the undersirable chattering in the VS/SM system). Furthermore, the simulations in Figure 5 prove that the system converges to the optimal operation point and stabilizes the system inside the observer.

## 5. Experimental Results

**Figure 10:**
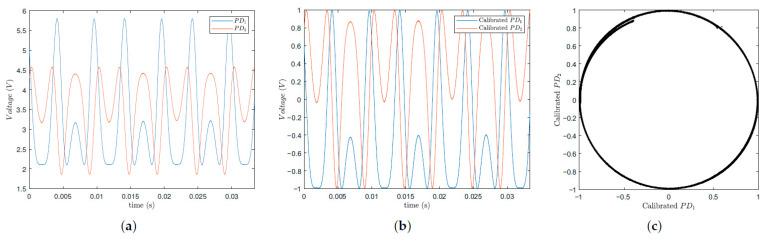
Normalization process. (**a**) Photo-detected signals. (**b**) Normalized photo-detected signals. (**c**) Lissajous figure of the normalized photo-detected signals.

This section presents the experimental results obtained using the experimental setup described earlier. In Figure 10, the results of the photodetected signal normalization process are presented. For this process, the actuator must exhibit a displacement of at least 2π. To achieve this, it was subjected to a sinusoidal signal of 100 HZ with an amplitude of 20 V. Data were collected with the NI BNC 2090A data acquisition board at a sampling frequency of 300 kHz. Since the relevant information lies in the signal phase, normalization is required. This process adjusts the signal to the range of −1 to 1 ($-1\leq PD_{1\;normalized},\;PD_{2\;normalized}\leq 1$), leaving only the cosine and sine terms from Equations (1) and (2). In Figure 10a, it is evident that the raw signal amplitude is greater than 1 and is not centered at zero. Thus, the interferometric signal is adjusted by subtracting its mean value and dividing by its amplitude to achieve unitary scaling and zero centering (($PD_{normalized}=\left(PD-mean\left(PD\right)\right)/\left(\left(max\left(PD\right)-min\left(PD\right)\right)/2\right)$). So, to perform the normalization shown in Figure 10b, the interferometric signal $PD_{1}$ had a mean value of 3.953 V and an amplitude of 1.855 V. Therefore, the normalized signal is given by $PD_{1\;normalized}=\left(PD_{1}-3.953\right)/1.855$ V. For the second signal, it is given by $PD_{2\;normalized}=\left(PD_{2}-3.217\right)/1.3627$ V. After applying this operation to both interferometric raw signals, the resulting normalized signals are $PD_{1\;normalized}=\cos\left(\Delta \phi \left(t\right)+\phi_{0}\left(t\right)\right)$; since the signals are phase-shifted by π/2, it can be concluded that, $PD_{2\;normalized}=\sin\left(\Delta \phi \left(t\right)+\phi_{0}\left(t\right)\right)$, in accordance with Figure 10b. When two signals with equal frequency and amplitude are phase shifted by π/2, their Lissajous figure is a circle. Since the amplitude is unitary, the circle’s radius is also 1. As expected, the plot of PD_1_ normalized versus PD_2_ normalized yields the circular Lissajous figure, as shown in Figure 10c.

The fact that the Lissajous plot shown in Figure 10c presents a portion in the upper-left region that does not overlap with the larger circle is intentional; this is in order to make it evident that—although our interferometer operates in open-loop mode (and is therefore susceptible to the occurrence of signal fading)—the proposed T-S fuzzy technique is immune to this problem and performs the detection of the desired phase accurately. In summary, since the interferometer operates in an open loop, temperature fluctuations and environmental mechanical vibrations act on the sensor; these drifts influence the optical path difference between the interferometer arms, causing the quasi-static phase shift $\phi_{0}\left(t\right)$ in Equation (1) to vary in time; this disturbs the periodicity of the quadrature phase photo-detected signal samples (see Figure 10), which in turn disturbs the radius of the circular orbit of the Lissajous figure over time. Despite this, the observer system of the new proposed method is able to compensate this variation in the low-frequency term $\phi_{0}\left(t\right)$ in an active manner (using the fuzzy T-S control system), performing the demodulation of the optical phase of interest correctly.

**Figure 11:**
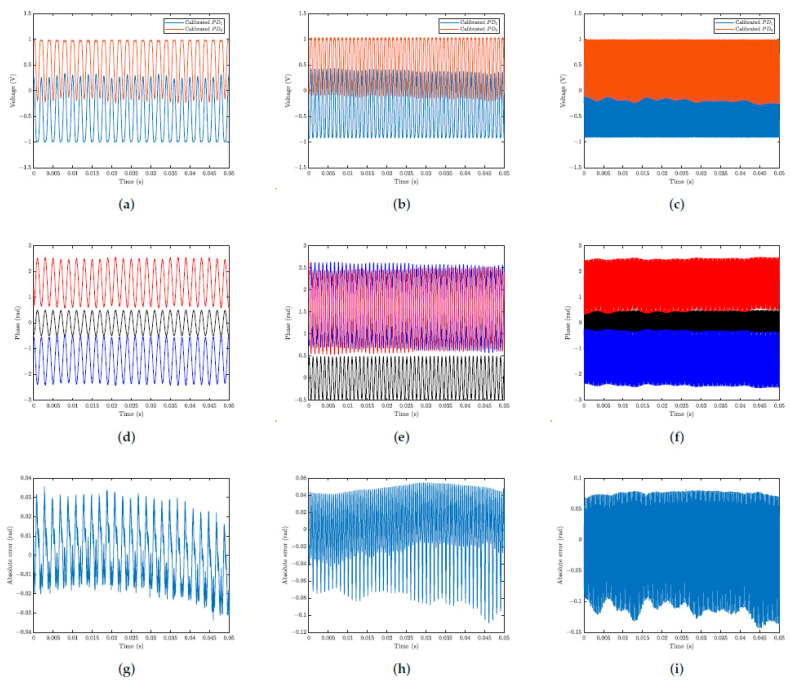
Experimental results by using the T-S fuzzy and arc tangent methods: input signal (in black color −); T-S fuzzy output (in red color −); arc tangent output (in blue color −). (**a**) Normalized interferometry signals for a 500 Hz input. (**b**) Normalized interferometry signals for a 1000 Hz input. (**c**) Normalized interferometry signals for a 4000 Hz input. (**d**) Demodulated signals for a 500 Hz input. (**e**) Demodulated signals for a 1000 Hz input. (**f**) Demodulated signals for a 4000 Hz input. (**g**) Absolute error between demodulated signals at 500 Hz input. (**h**) Absolute error between demodulated signals at 1000 Hz input. (**i**) Absolute error between demodulated signals at 4000 Hz input.

In Figure 11, the results for optical phase demodulated of the actuator over time are presented. For comparison, the well-established arc tangent demodulation method is used alongside the proposed method. (It is important to emphasize that the arc tangent method uses the signals measured from photodetectors $PD_{1}$ and $PD_{2}$, the output signals of the open loop interferometer. Therefore, it is a fully passive interferometry detection technique.) The classical arc tangent method detects the sum Δϕ(t)+ϕ(t) and, therefore, a high-pass filter with a cutoff at approximately 20 Hz must be used, in order to avoid oscillations in Δϕ(t) due to spurious variations in $\phi_{0}\left(t\right)$. The *filtfilt* function (from MATLAB) was used, which performs the phase correction of the filtered signal. For lower signal distortion, a 100th order FIR filter using the hamming window was used. The signals were normalized before the displacement measurements were performed. The data acquisition sampling frequency was 300 kHz, and the actuator was subjected to a signal with an amplitude of 4.8 V, with frequencies of 500, 1000, and 4000 Hz. The first column in Figure 11 shows the results for 500 Hz (Figure 11a,d,g), the second column for 1000 Hz (Figure 11b,e,h), and the third column for 4000 Hz (Figure 11c,f,i). The first row represents the normalized interferometric signals (Figure 11a–c). The second row shows the normalized input signal (in black color), the optical phase demodulated by the T-S fuzzy method (in red color), and the arc tangent method (in blue color) (Figure 11d–f). The third row shows the absolute error between demodulated signals (Figure 11g–i).

In Figure 11, in the second row, for all the frequencies used (500 Hz, 1000 Hz, and 4000 Hz), it is observed that the signals demodulated by the T-S fuzzy method stabilize at π/2, as predicted by the system and simulations (Figure 5). Depending on the relative position of the two photodiodes, PD1 and PD2 on the fringe pattern, positioned on two consecutive fringes, quadrature signals out of phase by +π/2 rad or −π/2 rad can be generated. This type of concern does not cause problems in most practical applications in interferometry; however, when such an effect is relevant, the choice of the two interference fringes (on which the photodiodes must be positioned) should be a matter of attention. In turn, the fuzzy T-S method does not use trigonometric functions (such as the arc tangent) to perform the optical phase shift demodulation and, therefore, does not present this drawback. From Figure 11d–f shown above, it can be seen that, by multiplying the results obtained by the arc tangent method by −1, there is practically an overlap of these results with those obtained by the T-S fuzzy method. Using the arc tangent method as the reference standard, the discrepancies in the fuzzy measurements were 0.0357 rad, 0.1094 rad, and 0.1437 rad for the 500 Hz, 1000 Hz, and 4000 Hz signals, respectively.

**Figure 12:**
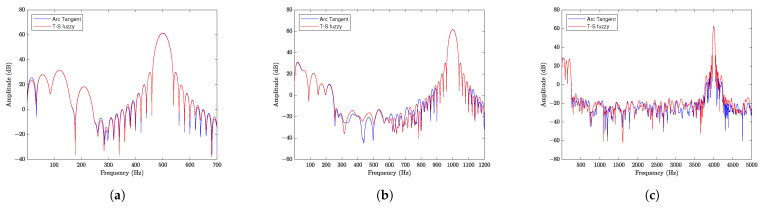
Spectral analysis of the demodulated signals. (**a**) Sine wave input at 500 Hz. (**b**) Sine wave input at 1000 Hz. (**c**) Sine wave input at 4000 Hz.

To verify the frequencies and reassess the amplitude exclusively at the Δϕ(t) frequency, a spectral analysis of the signals was conducted for each tested frequency. Figure 12 presents the spectral analyses: Figure 12a corresponds to the 500 Hz signal, Figure 12b pertains to the 1000 Hz signal, and finally, Figure 12c relates to the 4000 Hz signal. It is noteworthy that, unlike the simulated spectra shown in Figure 6, these experimental spectra correspond to the steady-state operation regime.

The spectral analyzes presented in Figure 12 were intentionally not adjusted. This highlights that the peak frequencies of the actuator’s input signal coincide with the two demodulation methods. It can also be observed that, for the T-S fuzzy method, low frequencies are attenuated; this result was expected, since the method aims to eliminate the effect of $\phi_{0}\left(t\right)$ on the measurement of optical phase shift. Just as good agreement was observed between results using the T-S fuzzy and arc tangent techniques (with proper signal inversion) when measured in the time domain, good agreement was also observed between the spectra measured at different frequencies.

**Figure 13:**
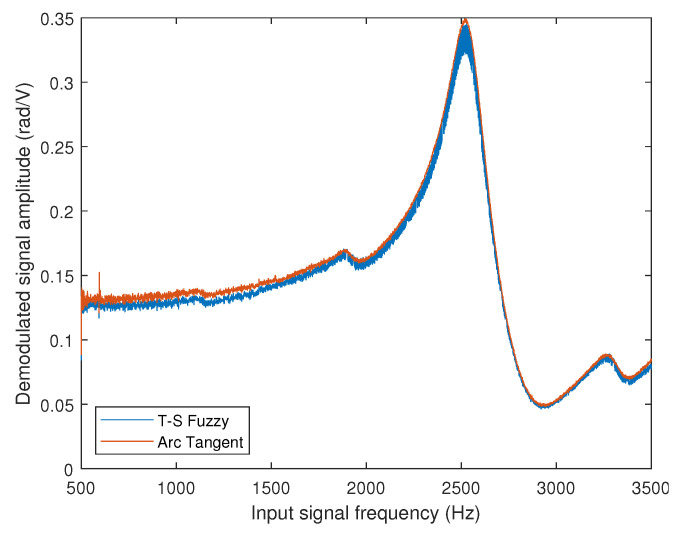
Frequency response of the commercial piezoelectric actuator (Control Technics).

In Figure 13, the frequency response of the commercial PZT actuator was obtained in the range of 500 to 3500 Hz with a step of 1 Hz. The measured phase shift was normalized by the voltage of the signal applied to the actuator. The procedure involved determining the frequency response using two demodulation methods: the T-S fuzzy method proposed in this work (blue line) and the arc tangent method used as a standard (red line).

Observing the processed data, a flat band (3 dB variation) from 500 to 1000 Hz can be observed, with the main resonance near 2518 Hz. The agreement between the two methods used is also observed, as both present approximately the same frequency response. We may conclude that the interferometry detection method with fuzzy T-S observer-based control is as good as the arc tangent method. The discrepancy between the two methods over the 3dB frequency bandwidth is less than 5.54%, with an average discrepancy of 1.72% over the band between 500 Hz and 3500 Hz.

## 6. Conclusions

In this work, we proposed a novel proof-of-concept method based on T-S fuzzy logic for the demodulation of interferometric signals. The authors think that the proposed procedure—considering the optical interferometry observer described by a T-S fuzzy model presented in (13)—opens new and important possibilities for designing powerful controllers for the interferometry area. Based on the classical concept of high-gain (HGA) interferometry proposed by [50], and by the modern nonlinear observer based on a VS/SM (variable structure and sliding mode) nonlinear control system proposed by [29], the authors reproduced the assignment of a closed-loop interferometer in LGA (low gain approach) mode [14]; however, an open-loop interferometer is used with an observer based on a nonlinear control strategy. The optical hardware is straightforward (a simple bulk Michelson interferometer), while the software runs a closed-loop observer based on T-S fuzzy nonlinear control. The new method does not have the undesirable problems of glitches or the interruption of the measurement caused by the reset circuits that were necessary in [50], nor the chattering problems present in [29]. The control method compensates for phase drifts caused by the environment and maintains the operating point at its unique equilibrium. The computational results indicate that the control method can effectively demodulate signals up to an amplitude of π/2 rad and with frequencies starting at 100 Hz (corresponding to a maximum error of 0.45%). The experimental results, through comparison with the arc tangent method, showed that the fuzzy T-S method can demodulate interferometric signals with discrepancies smaller than 0.0357 rad, 0.1094 rad, and 0.1437 rad for signals at 500 Hz, 1000 Hz, and 4000 Hz, respectively. Using this method, it was possible to determine the frequency response of a piezoelectric actuator, presenting practically the same result when compared to the important arc tangent method (with a discrepancy of less than 5.54% across the 3 dB frequency bandwidth). On the other hand, simulations showed that the T-S fuzzy method is more immune to noise effect than the arc tangent method. In fact, by inserting noise with SNR = 5 dB, the control system remained at its operating point, unlike the arc tangent method, which presented 2π phase jumps. The authors intend to apply modifications to the present fuzzy controller, specifically, using the high-gain approach (HGA) strategy. Our future work will focus on expanding the dynamic range of the method by modeling the system according to the HGA mode, through a nonlinear observer, similar to the one proposed by [29], however, using T-S fuzzy control instead of VS/SM control. 

## Data Availability

The data presented in this study are available on request from the corresponding author.

## Abbreviations

The following abbreviations are used in this manuscript:

AI	artificial intelligence
EFPI	extrinsic Fabry–Perot interferometer
FOCS	fiber optic current sensor
FOVT	Fiber optic voltage transformer
HGA	high-gain approach
LGA	low-gain approach
LMI	linear matrix inequality
LPF	low-pass filter
PDC	parallel-distributed compensation
PGC	phase-generated carrier
PID	proportional–integral–derivative
PSHS	parallel subsystem of hardware simulation
SHF	Structural Health Monitoring
T-S	Takagi–Sugeno
VS/SM	variable structure and sliding modes
WLI	white light interferometry
